# Influence of fear of COVID-19 on depression: The mediating effects of anxiety and the moderating effects of perceived social support and stress perception

**DOI:** 10.3389/fpsyg.2022.1005909

**Published:** 2023-01-09

**Authors:** Xiaoyu Li, Pengcheng Yang, Yanju Jiang, Dongdong Gao

**Affiliations:** ^1^School of Philosophy and Public Administration, Henan University, Kaifeng, China; ^2^Institute of Psychology and Behavior, Henan University, Kaifeng, China

**Keywords:** fear of COVID-19, depression, anxiety, perceived social support, stress perception

## Abstract

**Objective:**

Explore the influence of fear of COVID-19 on depression, with anxiety as a mediator and perceived social support and stress perception as moderates.

**Methods:**

From February to March 2020, 1,196 valid data were collected online through questionnaire by cluster sampling method. Fear of COVID-19 Questionnaire, the Patient Health Questionnaire 9-Item Scale (PHQ-9), the Generalized Anxiety Disorder 7-Item Scale (GAD-7), the Perceived Social Support Scale (PSSS) and the10-item Perceived Stress Scale (PSS-10) were used as the survey instrument, and the participants were female undergraduates from a liberal arts college of a Chinese university. Common method bias was assessed using Harman’s single-factor test in SPSS and confirmatory factor analysis in AMOS. The levels of participants’ anxiety, depression and perceived social support were described using frequency and percentage, Pearson Correlation test was used to measure the correlation between the variables. The PROCESS macro for SPSS (Model 1, Model 4, and Model 21) were applied to examine the mediating effect and moderating effect of the model.

**Results:**

Fear of COVID-19 can positively influence depression, anxiety plays a mediating role between fear of COVID-19 and depression, perceived social support negatively moderates the relationship between fear of COVID-19 and anxiety, and stress perception positively moderates the relationship between anxiety and depression. These five variables can form a moderated mediating effect model.

**Conclusion:**

Fear of COVID-19, anxiety and stress perception are risk factors for depression, perceived social support is a protective factor for depression. Reducing the fear of COVID-19, anxiety and stress perception and enhancing perceived social support are beneficial to reduce the level of depression.

## 1. Introduction

The outbreak of the novel coronavirus disease 2019 in December 2019 swept the world in a short time. On March 9, 2020, the World Health Organization (WHO) announced that COVID-19 has become a global pandemic (World Health Organization, 2020). The characteristics of the suddenness, severity, prevalence and uncertainty of the COVID-19 made people deeply fearful (Cooke et al., 2020; Wu et al., 2021). Fear of COVID-19 has led to a decline in individual mental health and an increase in negative emotions such as depression and anxiety (Li et al., 2020). Undergraduates are in adolescence, their psychological development is not very mature, and they are prone to psychological problems due to the pressure of learning and employment (Auerbach et al., 2016, 2018). Fear of COVID-19 has made the psychological problems of undergraduates who lack social experience and coping measures more serious (Pierce et al., 2020; Son et al., 2020).

Fear of COVID-19 has different effects on undergraduates of different genders and majors (Pieh et al., 2020; Prowse et al., 2021). Studies have shown that female are more sensitive to the external environment, and fear of COVID-19 is more likely to bring them psychological problems (Gao et al., 2020; Li et al., 2020; Wang et al., 2020; Xiao et al., 2020; Zhang et al., 2020). A meta-analysis study on college students’ fear of COVID-19 showed that the mean of Fear of COVID-19 Scale (FCV-19S) score in women (17.11, 95% CI: 16.59–17.64) was higher than that in men (15.21, 95% CI: 14.33–16.08). A meta-analysis of fear of COVID-19 on adults showed that the mean of Fear of COVID-19 Scale (FCV-19S) score in women (20.67, 95% CI: 18.62–22.73) was higher than that in men (18.21, 95% CI: 15.99–20.42). Moreover, the mean score in Asian women and men was 19.70 and 17.68 and in American women and men was 20.39 and 16.15 (Luo et al., 2021; Wang et al., 2022). COVID-19 has brought different effects on the mental health of undergraduates of different majors. Wei et al. (2020) investigated 1,580 undergraduates, the results showed that the scores of depression, neurasthenia, fear, compulsion anxiety and hypochondriasis factors of liberal arts, science and engineering, and medical undergraduates were statistically different. In terms of fear factors, liberal arts undergraduates scored higher than medical undergraduates. Wang et al. (2021) investigated 3,179 undergraduates and found that liberal arts undergraduates scored higher in GAD-7 than art and other categories undergraduates, and liberal arts undergraduates scored higher in PHQ-9 than science and engineering undergraduates. Min and Sun (2021) investigated 790 undergraduates and found that liberal arts undergraduates scored higher than science and engineering undergraduates in SCL-90. Since undergraduates of different genders and majors have different responses to the COVID-19, in order to make the research more targeted, this paper will explores the influence of fear of COVID-19 on female undergraduates majoring in liberal arts.

**Figure 1:**
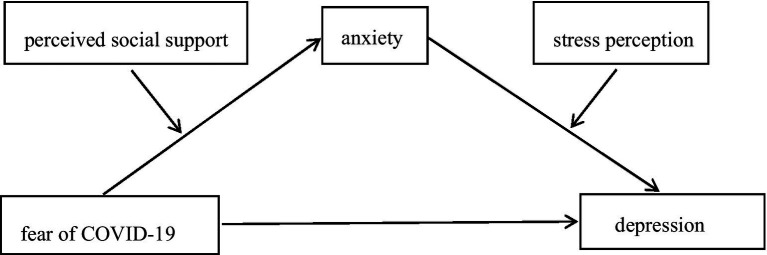
Hypothesized model.

Previous studies have shown that fear of COVID-19 has a positive influence on anxiety and depression, but few studies have paid attention to whether anxiety leads to depression or depression leads to anxiety during the COVID-19 pandemic. This study believes that fear of COVID-19 will lead to anxiety and depression at the same time, but anxiety can play a mediating role between fear of COVID-19 and depression, because during the COVID-19 epidemic, individuals can get less help, and anxiety will be transformed into depression in the case of helplessness, that is, individuals will have the experience of learned helplessness when dealing with the fear of COVID-19, which leads to depression. As an important protective factor, social support can reduce the negative influence of external stimuli on individuals. This study believes that perceived social support, as a moderating variable, affects the relationship between fear of COVID-19 and anxiety, that is, under the condition of low perceived social support, the relationship between fear of COVID-19 and anxiety is closer. The COVID-19 epidemic and other factors inevitably bring stress to individuals, but individuals have different perceptions of stress. Therefore, this study takes the stress perception as a moderating variable, and believes that the relationship between anxiety and depression is closer in the case of high stress perception. Due to the complexity of the environment, perceived social support and stress perception often coexist, and they jointly influence the relationship between fear of COVID-19 and anxiety and depression. Based on this, this study constructs a moderated mediating effect model that can answer not only “how” fear of COVID-19 influence depression, but also “when” this effect is stronger or weaker. The specific theoretical model is shown in Figure 1.

## 2. Theory and hypotheses

### 2.1. The influence of fear of COVID-19 on depression

Fear is a negative emotion that occurs when an individual perceives a threat to himself or his environment, it is associated with danger, injury or pain (Scalabrini et al., 2020). Humans are born to fear all sorts of different things, and COVID-19 has inevitably caused individual fear (Schimmenti et al., 2020). Undergraduates’ fear of COVID-19 has both direct and indirect factors. The direct factor is the concern about the influence of COVID-19 on their health, and the indirect factor is the concern about the influence of COVID-19 on their family, study, employment and future. According to the cognitive vulnerability theory of depression (Beck and Dozois, 2011), the fear of COVID-19 leads to individuals unable to view the COVID-19 epidemic correctly, exaggerating the direct and indirect negative influence of the COVID-19 epidemic on personal health and future development, and underestimating the individual’s coping ability, which leads to depression. Although scholars have different definitions of depression, they agree that “low mood” is the typical symptom of depression, and the core manifestation of depression is “anhedonia.” According to the hopeless theory of depression (Abramson et al., 1989), the fear brought by COVID-19 can arouse undergraduates’ attention to their health at the beginning, however, the accompanying insecurity and negative emotions can cause the individual to feel hopeless and helpless, which leads to depression. Several studies have shown that fear is an important risk factor for depression (Ying et al., 2014), the COVID-19 pandemic has led to increased levels of depression among undergraduates (Deng et al., 2020; Vahedian-Azimi et al., 2020). Therefore, the following hypothesis is proposed:

*H1*: Fear of COVID-19 positively influences depression.

### 2.2. The mediating effect of anxiety on the relationship between fear of COVID-19 and depression

Anxiety refers to the complex emotional state such as tension, uneasiness, sadness and trouble when individuals subjectively believe that there will be some adverse consequences or dangerous situations. The COVID-19 has threatened the health of undergraduates and their families, and their academic, employment and interpersonal interactions are affected (Hasan and Bao, 2020), all of these can make undergraduates anxious (Koçak et al., 2021; Mahmud et al., 2021). Several studies have shown that anxiety levels among undergraduates have increased during the COVID-19 pandemic (Bakioglu et al., 2021; Li et al., 2021; Zhan et al., 2021). Therefore, it can be speculated that the fear of COVID-19 has led to increased anxiety among undergraduates. Although there are similarities between anxiety and depression, there are obvious differences between them. The typical characteristic of anxiety is nervousness, while the typical characteristic of depression is hopelessness, helplessness and worthlessness caused by low mood (Beuke et al., 2003). When the individual’s anxiety cannot be relieved for a long time, or even continues to strengthen, it is possible to produce depression (Reinherz et al., 1989, 1993). Studies proved that 5-hydroxytryptamine in depression is significantly lower than that in anxiety disorder. The continuous spectrum of 5-hydroxytryptamine from normal to slightly reduced to moderately reduced seems to prove the development process from normal to anxiety to depression (Lou and Niu, 2009). According to the anxiety-depression continuum hypothesis (Sufka et al., 2006), the occurrence of anxiety and depression disorders has a clear chronological order, which can be roughly divided into three stages: (1) anxiety stage; (2) conversion stage of anxiety and depression; (3) depression stage (Liang et al., 2017). These two studies seem to suggest that anxiety causes depression. In the face of the negative influence of COVID-19, undergraduates will have anxiety such as nervous and worry at the beginning. However, as individuals have few active measures to take in the face of COVID-19, their anxiety cannot be alleviated, which will lead to depression (Faraci et al., 2022; Zhang et al., 2022). Therefore, the following hypothesis is proposed:

*H2*: Anxiety plays a mediating role between the fear of COVID-19 and depression.

### 2.3. The moderating effect of perceived social support on the relationship between fear of COVID-19 and anxiety

Both the main-effect model and the buffering model of social support believe that social support can relieve stress and improve mental health (Etzion, 1984; Cohen and Wills, 1985; Masten, 2001). Whether it directly increases individual happiness, reduces negative emotions such as anxiety and depression, or buffers the negative influence of negative events on individuals, social support will play a positive role (Sun et al., 2021). For individuals, it is very important to understand social support, because no matter how much material and spiritual support, if individuals think it has no effect on them, then these support will not be effective. During the COVID-19 epidemic, the role of perceived social support is particularly prominent due to the limited conditions (Lai et al., 2020; Szkody et al., 2021). When undergraduates perceive low levels of social support, fear of COVID-19 causes more anxiety. When undergraduates perceived higher levels of social support, although the anxiety increases as the fear of COVID-19 increases, the level of increase has decreased compared with the former. Therefore, the following hypothesis is proposed:

*H3*: Perceived social support plays a moderating role between the fear of COVID-19 and the anxiety, and the relationship between fear of COVID-19 and anxiety is stronger when perceived social support is low.

### 2.4. The moderating effect of stress perception on the relationship between anxiety and depression

Stress is an individual’s arousal state and nervous response to stimulation (Rehman et al., 2021). Stress perception is an individual’s cognition and evaluation of stress. From the cognitive evaluation theory (Lazarus, 1991, 1998), COVID-19 has affected everyone’s life and brought stress to everyone. However, different people have different subjective perceptions of COVID-19. Some people have a lot of stress when facing COVID-19, while others have less stress. Therefore, the same stressful event produces different perception of stress in each person. The fear of COVID-19 inevitably generates stress perception among undergraduates. At a high level of stress perception state, depression will increase quickly with the increase of anxiety. At the lower levels of stress perception state, although depression will increase with the increase of anxiety, the increased level has decreased compared with the former. Therefore, the following hypothesis is proposed:

*H4*: Stress perception plays a moderating role between the anxiety and the depression, and the relationship between the anxiety and the depression is stronger when stress perception is high.

### 2.5. A moderated mediating effect among the five variables

In previous part of the paper, we assumed that the fear of COVID-19 had a positive influence on depression, and that anxiety played a mediating role between the fear of COVID-19 and depression. The mediating model composed of these three variables was conducive to exploring “how” fear of COVID-19 influenced depression. In order to explore the “boundaries” and “conditions” of fear of COVID-19 influencing depression, it is assumed that perceived social support plays a moderating role between fear of COVID-19 and anxiety, and stress perception plays a moderating role between anxiety and depression. The moderating model is helpful to explore the influence of fear of COVID-19 on depression “when” is stronger or weaker. The relationship between fear of COVID-19, depression, anxiety, perceived social support and stress perception can further constitute a double-moderated mediating effect model, that is, perceived social support and stress perception jointly moderated the mediating effect of anxiety between fear of COVID-19 and depression. Perceived social support and stress perception can be combined into four modes: high perceived social support and high stress perception, high perceived social support and low stress perception, low perceived social support and high stress perception, low perceived social support and low stress perception. Since perceived social support is a protective factor and stress perception is a risk factor, this study infers that under the conditions of high perceived social support and low stress perception, fear of COVID-19 has less indirect influence on depression through anxiety; under the condition of low perceived social support and high stress perception, fear of COVID-19 has more indirect influence on depression through anxiety. Therefore, the following hypothesis is proposed:

*H5*: Fear of COVID-19, depression, anxiety, perceived social support and stress perception can form a double-moderated mediating effect model, perceived social support and stress perception jointly moderated the mediating effect of anxiety between fear of COVID-19 and depression.

## 3. Materials and methods

### 3.1. Population

According to the information released by the Ministry of Education of the People’s Republic of China on August 30, 2021, the number of Chinese undergraduate students in 2020 was 18,257,460, including 9,804,641 female students, accounting for 53.702%. The number of liberal arts students was 9,158,085, accounting for 50.161% (Ministry of Education of the People’s Republic of China, 2021). The estimated number of female liberal arts undergraduates in 2020 will be about 4.91 million. In order to make the study more targeted, we chose female undergraduate students of liberal arts as participants and use questionnaire survey method to collect data. The participants were female undergraduates from freshman to senior year in a liberal arts college of a university in China.

### 3.2. Data collection

This study collected data through questionnaire by cluster sampling method. The criteria for inclusion of the participants were: (1) female undergraduate, (2) major in liberal arts, and (3) voluntary participation in the study. The questionnaire was distributed from February to March 2020, due to the prevention and control of COVID-19 at that time, “Wenjuanxing (a widely used and professional questionnaire platform)” was selected for online testing. The confidentiality of the results was emphasized before the test, and the questionnaire was filled out voluntarily by the participants. A total of 1,213 questionnaires were distributed online, after collecting the questionnaire, review the answers, delete linear and wavy answers, and 1,196 valid questionnaires were collected. The exclusion criteria of the participants are mainly based on the answers to the questionnaire. The questionnaires with one of the following conditions are excluded: (1) the questionnaire answers in a straight line, and (2) the questionnaire answers in waves.

### 3.3. Demographic profile

Table 1 presents the demographic characteristics of the sample. Of the 1,196 participants, 212 (17.726%) were in grade one, 325 (27.174%) in grade two, 337 (28.177%) in grade three and 322 (26.923%) in grade four; 69 (5.769%) participants were aged 18 years and below, 203 (16.973%) were aged 19 years, 315 (26.338%) were aged 20 years, 301 (25.167%) were aged 21 years, 295 (24.666%) were aged 22 years, and 13 (1.087%) were aged 23 years and above; 935 (78.177%) participants majored in Chinese language and literature, 68 (5.686%) majored in Teaching Chinese to speakers of other language, 132 (11.037%) majored in Drama and film, and television literature, and 61 (5.100%) majored in Secretarial studies; 721 (60.284%) participants currently live in Henan province and 475 (39.716%) in other province (not Henan province); 651 (54.431%) participants’ family addresses were in urban district and 545 (45.569%) in county town.

**Table 1:** Demographic information of sample (*N* = 1,196).

Characteristics	Item	*n*	%
Grade	One	212	17.726
	Two	325	27.174
	Three	337	28.177
	four	322	26.923
Age	18 years and below	69	5.769
	19	203	16.973
	20	315	26.338
	21	301	25.167
	22	295	24.666
	23 years and above	13	1.087
Major	Chinese language and literature	935	78.177
	Teaching Chinese to speakers of other language	68	5.686
	Drama and film, and Television literature	132	11.037
	secretarial studies	61	5.1
Province	Henan Province	721	60.284
	Other provinces (not Henan Province)	475	39.716
Family	Urban district	651	54.431
Address	County town	545	45.569

### 3.4. Measurement

#### 3.4.1. The fear of COVID-19 questionnaire

The Fear of COVID-19 Questionnaire was used to measure the participants’ fear of COVID-19. It developed by ourselves according to the questionnaire preparation procedure (Wu, 2010), contained 4 items, each item was rated on a 5-point Likert scale, ranging from 1 to 5 (1 = strongly disagree, 5 = strongly agree), with higher scores indicating higher levels of fear. The contents of the 4 items are as follows: “I fear that I will infect COVID-19″, “I fear that my family will infect COVID-19″, “I fear that my relatives and friends will infect COVID-19″, “I fear that COVID-19 will have a huge influence on my future.” In this study, the Cronbach’s alpha of this questionnaire was 0.915, the composite reliability (CR) was 0.919, the average variance extracted (AVE) was 0.744.

#### 3.4.2. The patient health questionnaire9-item scale

The Patient Health Questionnaire9-Item Scale (PHQ-9) was used to measure the participants’ depressive. It contained 9 items, measure the frequency of symptoms in the last 2 weeks, each item was rated on a 4-level rating model (0 = none, 1 = less than half the time, 2 = more than half the time, 3 = almost every day), total scores range from 0 to 27, with higher scores indicating higher levels of depressive (0–4 = no depression, 5–9 = mild depression, 10–14 = moderate depression, 15–19 = moderately severe depression, 20–27 = severe depression). The sample items include “Little interest or pleasure in doing some things,” “Trouble concentrating on things, such as reading the newspaper or watching television.” A large number of academic researches have shown that PHQ-9 with good reliability and validity (Kroenke et al., 2001; Richardson et al., 2010; Hu et al., 2014). In this study, the Cronbach’s alpha of this questionnaire was 0.911, the composite reliability (CR) was 0.913, the average variance extracted (AVE) was 0.543.

#### 3.4.3. The generalized anxiety disorder7-item scale

The Generalized Anxiety Disorder7-Item Scale (GAD-7) was used to measure the participants’ anxiety. It contained 7 items, measure the frequency of symptoms in the last 2 weeks, each item was rated on a 4-level rating model (0 = not at all, 1 = several days, 2 = more than half the days, 3 = nearly every day), total scores range from 0 to 21, with higher scores indicating higher levels of depressive (0–4 = no anxiety, 5–9 = mild anxiety, 10–14 = moderate anxiety, 15–21 = severe anxiety). The sample items include “Feeling nervous, anxious, or on edge,” “Trouble relaxing.” A large number of academic researches have shown that GAD-7 with good reliability and validity (Spitzer et al., 2006; Löwe et al., 2008; Qu and Sheng, 2015). In this study, the Cronbach’s alpha of this questionnaire was 0.937, the composite reliability (CR) was 0.938, the average variance extracted (AVE) was 0.685.

#### 3.4.4. The perceived social support scale

The perceived social support scale (PSSS) was used to measure the participants’ social support. It developed by Jiang (1999), which referred to the scale developed by Zimet et al. (1988). It contained 12 items, each item was rated on a 5-point Likert scale, ranging from 1 to 5 (1 = strongly disagree, 5 = strongly agree), with higher scores indicating higher levels of social support. PSSS has three dimensions as family support, friend support, and special person support, each dimension involves 4 items. The sample items include “My family really tries to help me,” “I have friends with whom I can share my joys and sorrows.” Academic research has shown that PSSS with good reliability and validity (Chen et al., 2018; De Maria et al., 2018). In this study, the Cronbach’s alpha of this questionnaire was 0.961, the composite reliability (CR) was 0.960, the average variance extracted (AVE) was 0.669.

#### 3.4.5. The 10-item perceived stress scale

The10-item perceived stress scale (PSS-10) was used to measure the participants’ stress perception. It contained ten items (six positively items, 1, 2, 3, 6, 9, 10; and four negatively items 4, 5, 7, 8), measure the stress the participants felt in the past month, each item was rated on a 5-point Likert scale, ranging from 0 to 4 (0 = never, 1 = occasionally, 2 = sometimes, 3 = often, 4 = always), total scores range from 0 to 40, with higher scores indicating higher levels of perceived stress (0–13 = low pressure level, 14–19 = medium pressure level, and 20–40 = high pressure level). The sample items include “In the last month, how often have you felt that things were going your way,” “In the last month, how often have you found that you could not cope with all the things that you had to do.” Academic research has shown that PSS-10 with good reliability and validity (Cohen et al., 1983; Wang et al., 2015; Kaya et al., 2017). In this study, the Cronbach’s alpha of this questionnaire was 0.854, the composite reliability (CR) was 0.848, the average variance extracted (AVE) was 0.395.

### 3.5. Statistical analysis

Data analyses were conducted using SPSS23.0 and AMOS23.0 (Wu, 2009, 2010), all comparisons were two-tailed, and *p*-values < 0.05were considered statistically significant. First, to establish the validity of the data, common method bias was assessed using Harman’s single-factor test in SPSS23.0 and confirmatory factor analysis in AMOS23.0. Second, descriptive analyses were conducted, participants’ sociodemographic characteristics and the levels of anxiety, depression and stress perception were described using frequency and percentage, Pearson Correlation test was used to measure the correlation between the variables. Third, hypothesis test were conducted using regression analysis and Hayes’ PROCESS macro for SPSS; to explore the direct effect of the fear of COVID-19 on depression by regression analysis; Model 4 of PROCESS was employed to test whether anxiety mediated the effect of the fear of COVID-19 on depression; Model 1 of PROCESS was employed to test whether perceived social support moderated the effect of the fear of COVID-19 on anxiety and stress perception moderated the effect of anxiety on depression; Model 21 of PROCESS was employed to test the double-moderated mediating effect model composed of five variables such as fear of COVID-19, depression, anxiety, perceived social support and stress perception (Hayes, 2013). In addition, mediating effect and moderating effect analyses were tested using non-parametric bootstrapping methods, the 95% confidence interval produced by the bootstrapping procedure was examined and if zero was not included within the confidence interval, the effect was considered significant (Preacher and Hayes, 2004; Preacher et al., 2007; Hayes, 2009).

## 4. Research results

### 4.1. Validity and reliability

The questionnaire used in this study, except for the fear of COVID-19 questionnaire which is self-compiled, the other four questionnaires (PHQ-9, GAD-7, PSSS, and PSS-10) are frequently used in psychological research. The Patient Health Questionnaire9-Item Scale (PHQ-9), the Generalized Anxiety Disorder7-Item Scale (GAD-7), the Perceived Social Support Scale (PSSS) and the 10-item perceived stress scale (PSS-10) have good reliability and validity under different cultural backgrounds and are widely used by researchers in various countries.

In this study, the Cronbach’s alpha of all questionnaires was higher than 0.85, CR was higher than 0.84, and AVE was higher than 0.5 except for one questionnaire which was 0.395. The standard value of Cronbach’s a is generally supposed to be 0.70 and higher (Rasool et al., 2019). According to Fornell and Larcker suggested, CR should exceed 0.6, and AVE should exceed 0.5 under ideal condition (Fornell and Larcker, 1981), while 0.36–0.5 are acceptable (Yin, 2018; Zhang and Zheng, 2021). So the Cronbach’s alpha, CR and AVE of the questionnaires in this study all meet the standard.

### 4.2. Measure results of PHQ-9, GAD-7, and PSS-10

Measure results of PHQ-9, GAD-7, and PSS-10 are presented in Table 2. Among 1,196 participants, PHQ-9 was used to measure depression, and the prevalence of “no depression (0–4)“was 38.796%, “mild depression (5–9)“was 41.388%, “moderate depression (10–14)“was 12.709%, “moderately severe depression (15–19)“was 5.100%, “severe depression (20–27)“was 2.007%. GAD-7 was used to measure anxiety, and the prevalence of “no anxiety (0–4)“was 49.080%, “mild anxiety (5–9)“was 40.886%, “moderate anxiety (10–14)“was 7.609%, “severe anxiety (15–21)“was 2.425%. PSS-10 was used to measure stress perception, and the prevalence of “a low-pressure level (0–13)“was 24.498%, “medium pressure level (14–19)“was 37.291%, “high-pressure level (20–40)“was 38.211%.

**Table 2:** Measure results of PHQ-9, GAD-7, and PSS-10.

Variable	Questionnaire	Score	Content	*n*	%
Depression	PHQ-9	0–4	No depression	464	38.796
		5–9	Mild depression	495	41.388
		10–14	Moderate depression	152	12.709
		15–19	Moderately severe depression	61	5.100
		20–27	Severe depression	24	2.007
Anxiety	GAD-7	0–4	No anxiety	587	49.080
		5–9	Mild anxiety	489	40.886
		10–14	Moderate anxiety	91	7.609
		15–21	Severe anxiety	29	2.425
Stress perception	PSS-10	0–13	Low pressure level	293	24.498
		14–19	Medium pressure level	446	37.291
		20–40	High pressure level	457	38.211

PHQ-9, the Patient Health Questionnaire9-Item Scale; GAD-7, the Generalized Anxiety Disorder7-Item Scale; PSS-10, the10-item perceived stress scale.

### 4.3. Descriptive statistics and correlation analysis

Means, standard deviations and correlations of the variables used in the analysis are presented in Table 3. The results reveal that the fear of COVID-19 is significantly positively correlated with depression (*r* = 0.268, *p* < 0.01), anxiety (*r* = 0.312, *p* < 0.01), perceived social support (*r* = 0.095, *p* < 0.01), and stress perception (*r* = 0.300, *p* < 0.01). Depression is significantly positively correlated with anxiety (*r* = 0.783, *p* < 0.01), stress perception (*r* = 0.641, *p* < 0.01) and significantly negatively correlated with perceived social support (*r* = −0.264, *p* < 0.01). Anxiety is significantly negatively correlated with perceived social support (*r* = −0.233, *p* < 0.01) and significantly positively correlated with stress perception (*r* = 0.648, *p* < 0.01). Perceived social support is significantly negatively correlated with stress perception (*r* = −0.336, *p* < 0.01).

**Table 3:** Mean, standard deviation, and correlation analysis results of each variable.

Variable	*M*	SD	1	2	3	4	5
1. FC	13.703	4.453	1				
2. Depression	6.432	5.095	0.268^**^	1			
3. Anxiety	4.902	4.083	0.312^**^	0.783^**^	1		
4. PSS	46.273	10.313	0.095^**^	−0.264^**^	−0.233^**^	1	
5. SP	17.243	5.770	0.300^**^	0.641^**^	0.648^**^	−0.336^**^	1

^**^p < 0.01; *M*, mean; SD, standard deviation; FC, fear of COVID-19; PSS, perceived social support; SP, stress perception.

### 4.4. The test of common method bias

In order to control the bias effect of common methods, the questionnaires with good reliability and validity were used as the measuring instruments. In the test process, the confidentiality of the results was emphasized, and some questionnaire items were scored using the reverse scoring method.

Harman single factor test was used to examine the common method (Podsakoff et al., 2003; Zhou and Long, 2004). The results of the exploratory factor analysis showed that the number of factors without rotation was greater than 1, and the variance interpretation percentage of the first principal component was 33.062%, less than 40%. This study also used confirmatory factor analysis to examine the common method bias in AMOS. Set the number of common factors as 1, and incorporate all items of the questionnaire into the same common factor for confirmatory factor analysis. The fitting index was as follows: *χ*^2^/*df* = 32.043, RMSEA = 0.161, SRMR = 0.208, TLI = 0.371, CFI = 0.402, the results show that the data fitting index is not good. According to the above research, it can be inferred that there was not significant common method bias in the measurement.

### 4.5. Hypothesis testing

The input method was used for linear regression analysis in SPSS, and the results revealed that the fear of COVID-19 positively influences depression (*B* = 0.306, SE = 0.032, *p <* 0.001). Therefore, Hypothesis 1 was verified.

In SPSS, the PROCESS macro was used to select Model 4 provided by Hayes for mediating effect analysis. As shown in Table 4, anxiety is a mediating variable, fear of COVID-19 is an independent variable, and depression is a dependent variable. Fear of COVID-19 can positively influences anxiety (*B* = 0.312, SE = 0.027, *p* < 0.001), anxiety can positively influences depression (*B* = 0.774, SE = 0.019, *p* < 0.001), fear of COVID-19 cannot positively influences depression (*B* = 0.026, SE = 0.019, *p* > 0.05), indirect effect is 0.242 (BootSE = 0.024, BootCI [0.196,0.290]), the mediating effect accounts for 90.299% of the total effect. Because anxiety has a significant mediating effect between fear of COVID-19 and depression, hypothesis 2 is verified.

**Table 4:** Mediating effect test.

	*B*	SE	*t*	*p*	LLCI	ULCI
FC → anxiety	0.312	0.027	11.356	0.000	0.258	0.366
Anxiety → depression	0.774	0.019	40.852	0.000	0.737	0.812
FC → depression	0.026	0.019	1.374	0.170	−0.011	0.063
	Effect					
Total effect	0.268	0.028	9.606	0.000	0.213	0.323
Direct effect	0.026	0.019	1.374	0.170	−0.011	0.063
	Effect	BootSE			BootLLCI	BootULCI
Indirect effect	0.242	0.024			0.196	0.290

B, unstandardized coefficients; SE, standard error; *t*, independent sample *t*-test; LLCI, the lowest value of the confidence interval; ULCI, the highest value of the confidence interval; FC, fear of COVID-19.

**Figure 2:**
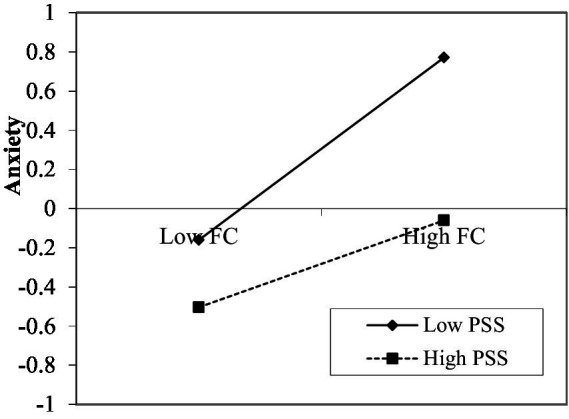
Moderating effect of PSS on the relationship between FC and anxiety. FC, fear of COVID-19; PSS, perceived social support.

**Figure 3:**
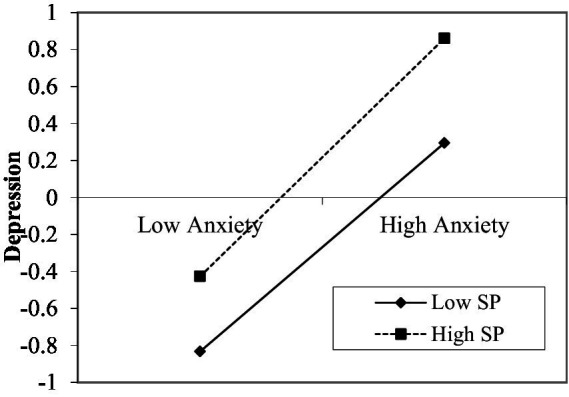
Moderating effect of SP on the relationship between anxiety and depression. SP, stress perception.

In SPSS, the PROCESS macro was used to select Model 1 provided by Hayes for moderating effect analysis. It can be seen from Table 5, when perceived social support is a moderating variable, the interaction coefficient between the fear of COVID-19 and perceived social support is significant (*B* = –0.122, SE = 0.023, *p <* 0.001), it shows that perceived social support plays a moderating role. Therefore, hypothesis 3 has been verified. In order to better understand the moderating effect of perceived social support between the fear of COVID-19 and anxiety, the perceived social support is taken at three different levels according to the average value and the average value plus or minus a standard deviation (*M*–1SD, *M*, *M* + 1SD). When the level of perceived social support is low (*M*–1SD), anxiety will increase by 0.466 standard deviations for every 1 standard deviation increase in fear of COVID-19.When the level of perceived social support is high (*M* + 1SD), anxiety will increase by 0.222 standard deviation for every 1 standard deviation increase in fear of COVID-19.This suggests that when the perceived social support level is low, the correlation between fear of COVID-19 and anxiety is closer. The simple slope analysis diagram of fear of COVID-19 and anxiety drawn with perceived social support as the moderating variable reflects the same rule (Figure 2). It can be seen from Table 5, when stress perception is a moderating variable, the interaction coefficient between the anxiety and stress perception is significant (*B* = 0.040, SE = 0.014, *p <* 0.01), it shows that stress perception plays a moderating role. Therefore, hypothesis 4 has been verified. In order to better understand the moderating effect of stress perception between the anxiety and depression, the stress perception is taken at three different levels according to the average value and the average value plus or minus a standard deviation (*M*–1SD, *M*, *M* + 1SD). When the level of stress perception is low (*M*–1SD), depression will increase by 0.564 standard deviations for every 1 standard deviation increase in anxiety. When the level of stress perception is high (*M* + 1SD), depression will increase by 0.644 standard deviation for every 1 standard deviation increase in anxiety. This suggests that when the stress perception level is high, the correlation between anxiety and depression is closer. The simple slope analysis diagram of anxiety and depression drawn with stress perception as the moderating variable reflects the same rule (Figure 3).

**Table 5:** Moderating effect test.

	*B*	SE	*t*	*p*	LLCI	ULCI
FC → anxiety	0.344	0.026	13.099	0.000	0.293	0.396
PSS → anxiety	−0.294	0.027	−10.975	0.000	−0.346	−0.241
FC × PSS	−0.122	0.023	−5.364	0.000	−0.167	−0.078
	Effect					
PSS (*M*–1)	0.466	0.036	13.104	0.000	0.396	0.536
PSS (*M*)	0.344	0.026	13.099	0.000	0.293	0.396
PSS(*M* + 1)	0.222	0.034	6.537	0.000	0.155	0.288
	*B*					
Anxiety → depression	0.604	0.025	24.503	0.000	0.556	0.652
SP → depression	0.243	0.023	10.568	0.000	0.198	0.288
Anxiety × SP	0.040	0.014	2.950	0.003	0.014	0.067
	Effect					
SP (*M*–1)	0.564	0.033	17.303	0.000	0.500	0.628
SP (*M*)	0.604	0.025	24.503	0.000	0.556	0.652
SP (*M* + 1)	0.644	0.023	28.065	0.000	0.599	0.689

B, unstandardized coefficients; SE, standard error; *t*, independent sample *t*-test; LLCI, the lowest value of the confidence interval; ULCI, the highest value of the confidence interval; FC, fear of COVID-19, PSS, perceived social support; SP, stress perception, *M*, mean.

In order to better explore the relationship between the five variables, in SPSS, we use the PROCESS macro developed by Hayes and choose model21 to test the moderated mediating effect. According to Table 6, both perceived social support and stress perception jointly moderated the mediating effect of anxiety, but the direction and magnitude of the moderating effect were different. When the perceived social support level was low (*M*–1) and the stress perception level was high (*M* + 1), the indirect effect was the largest, reaching 0.300. When perceived social support level was high (*M* + 1) and stress perception level was low (*M*-1), the indirect effect was smallest, reaching 0.125.

**Table 6:** Mediating effects of anxiety on different moderated levels of PSS and SP.

PSS	SP	Effect	BootSE	BootLLCI	BootULCI
*M*–1SD	*M*–1SD	0.262	0.030	0.204	0.321
*M*–1SD	*M*	0.281	0.030	0.223	0.340
*M*–1SD	*M* + 1SD	0.300	0.032	0.238	0.362
*M*	*M*–1SD	0.194	0.021	0.154	0.237
*M*	*M*	0.207	0.021	0.169	0.250
*M*	*M* + 1SD	0.221	0.022	0.180	0.265
*M* + 1SD	*M*–1SD	0.125	0.023	0.083	0.172
*M* + 1SD	*M*	0.134	0.023	0.090	0.180
*M* + 1SD	*M* + 1SD	0.143	0.024	0.096	0.191

PSS, perceived social support; SP, stress perception; SE, standard error; LLCI, the lowest value of the confidence interval; ULCI, the highest value of the confidence interval; M, mean; SD, standard deviation.

## 5. Discussion

The purpose of this paper is to explore the mechanism and boundary conditions of the influence of fear of COVID-19 on depression, this study constructed a moderated mediating effect model with anxiety as the mediating variable and perceived social support and stress perception as the moderating variable. The results showed that fear of COVID-19 not only directly affected depression, but also indirectly affected depression through anxiety; the first half of the mediation pathway was moderated by perceived social support and the second half by stress perception. Perceived social support and stress perception jointly moderated the mediating effect of anxiety between the fear of COVID-19 and depression, and this effect is the smallest when the level of perceived social support is high and the level of stress perception is low, and the largest when the level of social perceived support is low and the level of stress perception is high.

### 5.1. Theoretical implications

First, proved that fear of COVID-19 can positively predict depression and anxiety, enrich the related research of depression and anxiety. The cognitive vulnerability theory of depression believes that the individual’s wrong and distorted cognitive concept of things leads to the emergence of depression (Beck and Dozois, 2011). Fear of COVID-19 made individuals unable to correctly understand and evaluate themselves and the situation, which leads to depression. The hopeless theory of depression believes that the negative explanation and attribution model lead to individual despair (Abramson et al., 1989), which leads to depression. When individuals are afraid of COVID-19, they are more likely to have feelings of helplessness and hopelessness, and this negative emotion leads to depression. Anxiety is a negative emotional state generated by an individual to a stimulus that may pose a threat. Fear of COVID-19 itself is a negative stimulus, which will inevitably increase individual anxiety. Therefore, this study believes that fear of COVID-19 can positively influence depression and anxiety, which is consistent with the conclusions of previous studies (Huang and Zhao, 2020; Warren et al., 2021).

Second, discussed the mediating effect of anxiety, and deepened the understanding of the relationship between anxiety and depression. In previous studies, anxiety and depression were often studied as outcome variables, and less explored who was the independent variable and who was the dependent variable between them. The main reason is that anxiety and depression are highly correlated, and even “different diseases have the same origin,” so it is difficult to clarify the relationship between them. Whether it is a normal emotional reaction or a psychopathological state, anxiety and depression are both negative experiences and often occur together, which shows the complexity of the relationship between anxiety and depression (Brady and Kendall, 1992). Scholars generally divide the relationship between anxiety and depression into three categories (Liang et al., 2017). The first category is continuous spectrum theory. It is believed that anxiety and depression are different manifestations of the same disease. With the aggravation of the original disease and the continuation of the course of the disease, anxiety may be secondary to depression, and depression may also be secondary to anxiety. The second category is dichotomous theory. It is believed that anxiety and depression have different causes, different manifestations and different treatment methods, which are two different forms of emotional states and mental diseases. At present, this is the view accepted by most scholars. The third category is the theory of comorbidity. It is believed that when anxiety and depression coexist, they are independent disease entities different from anxiety and depression, and they have the same biological basis and similar clinical manifestations (Yuan et al., 2001; Colleen et al., 2014). This study believes that anxiety and depression are two different negative emotional states or symptoms, so during the COVID-19, it is more meaningful to explore the relationship between them. The fear of COVID-19 has triggered the anxiety of undergraduates (Ye et al., 2020), and during the epidemic, there are fewer ways and channels to alleviate or eliminate anxiety than before. In this case, undergraduates are more likely to have a sense of helplessness and hopelessness, which will lead to depression (Abramson et al., 1978; Faraci et al., 2022). This proves that anxiety plays a mediating role between fear of COVID-19 and depression.

Third, discussed the protective role of perceived social support, and supported the main-effect model and the buffering model of social support. Both the main effect model and the buffer model of social support believe that social support is an important protective factor for individual mental health (Cohen and Wills, 1985; Ratanasiripong, 2012; Tambag et al., 2018), especially during the COVID-19 pandemic, social support is even more important. Social support itself is the combination of objective and subjective products, no matter how much objective material support, if individuals do not understand, then it will not work. During the COVID-19 pandemic, due to the limitation of conditions, all kinds of actual material support are less than usual. Therefore, it is more appropriate to use perceived social support as the research index and measurement tool. For undergraduates with a low level of perceived social support, their anxiety increased significantly with the increase of fear of COVID-19, because the COVID-19 had a tremendous influence on their study and life. When they feel a low level of social support, they will feel more isolated and helpless, resulting in a high level of anxiety. For undergraduates with a higher level of perceived social support, their anxiety level will increase with the increase of fear of COVID-19, but compared with undergraduates with lower level of perceived social support, their anxiety level is not as high as the former. This is because undergraduates with high level of perceived social support will have negative emotions such as anxiety when facing the COVID-19, but when they think that they have more social support as the backing, their psychological tension and anxiety will be relieved, so their anxiety will not be so strong.

Fourth, discussed the risk role of stress perception, and deepen the understanding of cognitive evaluation theory. Stress perception is an individual’s subjective cognition and evaluation of stress events. According to Lazarus’s cognitive evaluation theory (Lazarus, 1991, 1998), for the same stress event, different individuals have different cognition and evaluation, so they feel different stress. Although the COVID-19 has brought stress to everyone (Ying et al., 2014), the stress perception by different individuals is not the same. Undergraduates with high stress perception feel more stress, so with the increase of anxiety, their depression level will increase rapidly. Undergraduates with low level of stress perception perceive less stress, with the increase of anxiety, although their depression level will also increase, compared with undergraduates with high level of stress perception, the increase rate of depression level has slowed down.

Fifth, explored the jointly moderating effect of perceived social support and stress perception, and enrich the related research of the dynamic effect model of social support theory. The dynamic effect model of social support theory holds that social support and stress will affect the physical and mental health of individuals at the same time (Monroe and Steiner, 1986), and the relationship between social support and stress is also influencing and interacting with the changes of external conditions. In this study, both perceived social support and stress perception moderated the mediating effect of anxiety, and the result shows that when the level of social support is low and the level of stress perception is high, fear of COVID-19 has the greatest influence on depression through the mediation of anxiety; When the level of social support is high and the level of stress perception is low, fear of COVID-19 has the least influence on depression through the mediation of anxiety.

### 5.2. Practical implications

This study may have the following implications in practice: First, during the COVID-19 pandemic, more care and help should be given to female liberal arts undergraduates. In this study, the results showed that in terms of depression, if divided by 5 points (Zhan et al., 2021; Zhang et al., 2022), the detection rate was 61.204%; In terms of anxiety, if divided by 5 points (Li et al., 2021), the detection rate was 50.920%. This shows that female liberal arts undergraduates have a relatively high detection rate of depression and anxiety. The data shown in a published article can be used as a comparison (Zhu et al., 2021), the study used the PHQ-9 and GAD-7 to survey medical staff and general public in a Chinese city from February to March 2020. The results show that in terms of depression, if divided by 5 points, the detection rate of medical staff was 33.398%, the detection rate of general public was 32.758%. In terms of anxiety, if divided by 5 points, the detection rate of medical staff was 38.668%, the detection rate of general public was 42.495%. It can be seen from the above comparison that the detection rate of both medical staff and general public is lower than that of female liberal arts undergraduates. Therefore, we believe that female liberal arts undergraduates need more care, support and help from society, school and family (Jing et al., 2021). Second, measures should be taken to reduce the fear of COVID-19 among undergraduates. Fear of COVID-19 is a risk factor for mental health, which can directly lead to the increase of depression and anxiety. It is necessary to let undergraduates learn relevant knowledge of epidemic prevention and control, and let them know that as long as they pay attention to protection, they can effectively reduce the risk of infection, so as to reduce the fear of COVID-19.Third, the anxiety of undergraduates should be reduced. Anxiety played a completely mediating role in this study, which shows that anxiety is more likely to lead to depression during the COVID-19 pandemic. We should reduce anxiety by guiding undergraduates to formulate reasonable learning and physical exercise plans and opening a psychological hotline. Fourth, let undergraduates perceived a higher level of social support. Family members should communicate more, so that undergraduates can feel the warmth of their families and the love of their parents. The school should communicate with undergraduates through various channels to make them realize that the school cares and supports them at all times. Fifth, we should reduce the stress perception level of undergraduates. Stress perception is the individual’s subjective understanding and evaluation of stress events. During the COVID-19 pandemic, we should reduce the stress events that may pose a threat to undergraduates, cultivate undergraduates’ positive mentality and healthy personality, and let them correctly deal with all kinds of stress.

## 6. Limitations and future research

The limitations of this study are mainly manifested in four aspects. First, the participants are only female students from one liberal arts college in one university in China, no male students, no undergraduates in other majors, the universality and representativeness of the sample are insufficient. In the future, male students, undergraduates in other majors and more universities in more provinces in China should be sampled to improve the representativeness of the results. Second, the relationship between variables is explained by cross-sectional research, and the distinction between causality is insufficient. Future studies should collect longitudinal data at different time points to further verify the relationship between variables. Third, the fear of COVID-19 Questionnaire in this study was developed by the author himself. Although it has good reliability and validity in this study, more studies are needed to verify it when it is popularized. Fourth, there is a complex relationship between anxiety and depression. This paper only studies the relationship between anxiety and depression with female students of liberal arts as participants in the context of COVID-19. More studies on the relationship between anxiety and depression need to be conducted with different groups as participants in different backgrounds, so as to better discuss the relationship between anxiety and depression.

## 7. Conclusion

This study constructed a moderated mediating effect model to explore the mechanism and boundary conditions of the influence of fear of COVID-19 on depression. The results were as follows: (1) Fear of COVID-19 positively influence depression. (2) Anxiety plays a mediating role between fear of COVID-19 and depression. (3) Perceived social support negatively moderates the relationship between fear of COVID-19 and anxiety, and stress perception positively moderates the relationship between anxiety and depression. (4) Perceived social support and stress perception jointly moderate the mediating effect of anxiety between fear of COVID-19 and depression. When the level of perceived social support is low and the level of stress perception is high, fear of COVID-19 has the greatest influence on depression through the mediating effect of anxiety. When the level of perceived social support is high and the level of stress perception is low, fear of COVID-19 has the least influence on depression through the mediating effect of anxiety.

## Data availability statement

The raw data supporting the conclusions of this article will be made available by the authors, without undue reservation.

## Ethics statement

The studies involving human participants were reviewed and approved by the Research Ethics Committee of the Institute of Psychology and Behavior, Henan University. All participants agreed to participate in the study. Written informed consent to participate in this study was provided by the participants’ legal guardian/next of kin.

## Author contributions

XL and DG designed the study and contributed to the acquisition and interpretation of data, XL and YJ did the literature review and wrote the research protocol. XL and PY drafted and revised the manuscript. XL and DG contributed to the revisions in depth for the manuscript. All authors contributed to and approved the final manuscript.

## Conflict of interest

The authors declare that the research was conducted in the absence of any commercial or financial relationships that could be construed as a potential conflict of interest.

## Publisher’s note

All claims expressed in this article are solely those of the authors and do not necessarily represent those of their affiliated organizations, or those of the publisher, the editors and the reviewers. Any product that may be evaluated in this article, or claim that may be made by its manufacturer, is not guaranteed or endorsed by the publisher.
