# Supplementary material for: Influence of fear of COVID-19 on depression: The mediating effects of anxiety and the moderating effects of perceived social support and stress perception
Source: Front Psychol. 2023 Jan 9;13:1005909. doi: 10.3389/fpsyg.2022.1005909 (PMC9869132; doi:10.3389/fpsyg.2022.1005909)
Supplement: Supplementary file 1 [file Appendix_1.pdf]

Appendix

The Fear of COVID-19 Questionnaire

1. I fear that I will infect COVID-19.

2. I fear that my family will infect COVID-19.

3. I fear that my relatives and friends will infect COVID-19.

4. I fear that COVID-19 will have a huge influence on my future.

The Patient Health Questionnaire9-Item Scale

1. Little interest or pleasure in doing some things.

2. Feeling down,depressed or hopeless.

3.Trouble failing asleep ,staying asleep,or sleeping too much.

4.Feeling tired or having little energy.

5.Poor appetite or overeating.

6.Feeling bad about yourself-or that you are a failure or your family down.

7. Trouble concentrating on things,such as reading the newspaper or watching television.

8.Moving or speaking so slowly that other people could have noticed.or ,the oppsite-being so fidgety or restless that you have been moving around a lot more than usual.

9. Thoughts that you would be better off dead or hurt yourself in some way

The Generalized Anxiety Disorder7-Item Scale

1.Feeling nervous, anxious, or on edge.

2.Not being able to stop or contral worrying.

3.Worrying too much about different things.

4.Trouble relaxing.

5.Being so restless that it’s hard to sit still.

6.Becoming easily annoyed or irritable.

7.Feeling afraid as if something awful might happen.

The Perceived Social Support Scale

1. There is a special person (such as teachers, classmates) who is around when Iam in need.

2. There is a special person(such as teachers, classmates) with whom I can sharemy joys and sorrows.

3. My family really tries to help me.

4. I get the emotional help and support I need frommy family.

5. I have a special person (such as teachers, classmates) who is a real source ofcomfort to me.

6. My friends really try to help me.

7. I can count on my friends when things go wrong.

8. I can talk about my problems with my family.

9. I have friends with whom I can share my joysand sorrows.

10. There is a special person(such as teachers, classmates) in my life who caresabout my feelings.

11. My family is willing to help me make decisions.

12. I can talk about my problems with my friends.

The10-item Perceived Stress Scale

1. How often have you been upset because of something that happened unexpectedly?

2. How often have you felt that you were unable to control the important things in your life?

3. How often have you felt nervous and “stressed”?

4. How often have you felt confident about your ability to handle your personal problems?

5. How often have you felt that things were going your way?

6. How often have you found that you could not cope with all the things that you had to do?

7. How often have you been able to control irritations in your life?

8. How often have you felt that you were on top of things?

9. How often have you been angered because of things that were outside of your control?

10. How often have you felt difficulties were piling up so high that you could not overcome them?
